# Investigating the Usage of Abiraterone in African American Men with Metastatic Prostate Cancer

**DOI:** 10.1007/s40615-025-02533-3

**Published:** 2025-06-24

**Authors:** Christine Ibilibor, Marieke Jones, Jeffrey Tomaszewski, Serge Ginzburg, Andres Correa, Robert Uzzo, Marc Smaldone, John Danella, Thomas J. Guzzo, Daniel Lee, Laurence Belkoff, Jeffrey Walker, Jay D. Raman, Adam Reese, Mihir S. Shah, Bruce Jacobs, Thomas Jang, Keith Kowalczyk, Meghan Smith

**Affiliations:** 1 Division of Urologic Oncology, Department of Urology, University of Virginia, 500 Ray C. Hunt Drive, Charlottesville, VA 22903 USA; 2 Department of Public Health Sciences, University of Virginia, Charlottesville, VA USA; 3 Division of Urology, Cooper University Health Care, Camden, NJ USA; 4 Department of Urology, Einstein Healthcare Network, Philadelphia, PA USA; 5 Division of Urologic Oncology, Fox Chase Cancer Center, Philadelphia, PA USA; 6 Department of Urology, Geisinger Health System, Danville, PA USA; 7 Division of Urology, University of Pennsylvania, Philadelphia, PA USA; 8 Division of Urology, MidLantic Urology/Main Line Health, Bala Cynwyd, PA USA; 9 Division of Urology, Penn State Health Milton S. Hershey Medical Center, Hershey, PA USA; 10 Department of Urology, Temple University, Philadelphia, PA USA; 11 Department of Urology, Thomas Jefferson University Hospital, Philadelphia, PA USA; 12 Department of Urology, University of Pittsburgh, Pittsburgh, PA USA; 13 Division of Urology, Rutgers Cancer Institute of New Jersey, New Brunswick, NJ USA; 14 Department of Urology, MedStar Health Georgetown, Washington, DC USA; 15 Health Care Improvement Foundation, Philadelphia, PA USA

**Keywords:** Prostate cancer, Metastatic disease, Anti-androgens, African Americans

## Abstract

**Background:**

African American men bear an unequal proportion of metastatic prostate cancer burden. The utilization of second-generation anti-androgens like abiraterone with androgen deprivation therapy (ADT) has become a standard therapy for metastatic prostate cancer. Thus, we aimed to examine the utilization of abiraterone with ADT in African American men with metastatic disease and determine whether disparities in its use exist.

**Methods:**

This is a retrospective study, using a multi-institutional regional collaborative prostate cancer database. We compared the use of ADT with abiraterone between African American men and non-African American men diagnosed with distantly metastatic prostate cancer from July 2015 to August 2022, using logistic regression and negative binomial regression.

**Results:**

We identified 201 men with metastatic prostate cancer and of those, 28% were African American men. African American men were younger (47% vs. 27.8%, ≤ 69 years, *p* = 0.002), and younger men experienced a longer time between diagnosis of metastatic disease and receipt of ADT with abiraterone compared to older men (mean 125 vs. 14 days, *p* = 0.038). While African American and non-African American men had similar rates of ADT use with abiraterone, African American men experienced longer times between documented metastatic disease and initiation of ADT with abiraterone (mean 187 vs. 79 days, *p* = 0.042).

**Conclusion:**

The initiation of ADT with abiraterone was delayed by 3 months in African American men. This discrepancy warrants an investigation of system level barriers to the timely initiation of abiraterone in African American men given its known oncologic benefits.

## Introduction

African American men are more likely to be diagnosed with metastatic prostate cancer than their non-African American counterparts, leading to rates of prostate cancer-specific mortality that are two to four times higher than those in other racial groups [1]. The unequal burden of metastatic prostate cancer that African American men bear in the USA and the associated disparities in prostate cancer care that they experience remain well documented particularly outside of equal access health systems [2]. Metastatic prostate cancer is primarily treated with androgen deprivation therapy (ADT) and second-generation anti-androgens are designed to augment the anti-tumor effects of ADT by either inhibiting prostatic and extra-prostatic testosterone biosynthesis or binding with a higher affinity to the androgen receptor to reduce tumor growth [3, 4]. Clinical trials have shown that the biochemical properties of second-generation anti-androgens like abiraterone, apalutamide, enzalutamide, and darolutamide used in combination with ADT lead to improvements in overall survival among men with metastatic prostate cancer [5]. As a result, ADT intensification with second-generation anti-androgens has become standard of care [6–10]. Thus, the use of agents such as abiraterone are being employed with ADT at increasing rates in oncologic practices as a result of seminal trials like LATITUDE [9, 11]. However, the utilization of ADT intensification remains understudied in African American men as they are often not included or comprised less than 2% of participants in metastatic prostate cancer clinical trials [6, 7, 9, 10]. Given the low representation of African American men in clinical trials, disparities in their oncologic outcomes have the potential to widen as new agents in the management of metastatic prostate cancer are developed [12]. As a result, our understanding of the usage of these agents in the African American with metastatic disease remains limited and can contribute to the persistently poorer oncologic outcomes in these men. Thus, we sought to examine the usage and timing of abiraterone in combination with ADT among African American men with distantly metastatic prostate cancer and their non-African American counterparts in a multi-institutional regional collaborative prostate cancer database. We utilized the Pennsylvania Urologic Regional Collaborative/Urologic Surgeons Comparing Outcomes Pursuing Excellence (PURC) database for this study due to the large geographic area it covers, and diversity in practice settings, giving it the ability to reflect real-world clinical practice.

## Materials and Methods

### Study Cohort

Data from the PURC database was used. The PURC database is a prospectively maintained quality improvement collaborative comprised of 14 academic and community urology practices in the Mid-Atlantic region, including 169 urologists. Practices in the PURC include private and academic institutions that cover a wide range of communities such as urban and rural settings. Only men diagnosed with clinically localized prostate cancer managed with surgery, radiation, or active surveillance were enrolled in the PURC database across participating practices. We performed a retrospective analysis on all patients in the PURC database who were subsequently diagnosed with distantly metastatic prostate cancer during the follow-up period as men with de novo metastatic disease were not included in the PURC database. This study was approved by the institutional review board at each participating institution, and informed consent was waived as the research involved no more than minimal risk to the participants. Patients with documented clinical staging between July 2015 (first year data abstraction into PURC began) and August 2022 (last year of available data for analysis) were included. This study followed the Strengthening the Reporting of Observational Studies in Epidemiology (STROBE) reporting guidelines [13].

### Study Outcomes

Abiraterone was the only recorded anti-androgen used in the PURC database. Thus, our primary outcome was the proportion of African American men with distantly metastatic disease receiving ADT in combination with abiraterone compared to non-African American men. The secondary outcome was time in days between change in clinical stage from localized to distantly metastatic disease (or M1 clinical stage) to the initiation of ADT with abiraterone. African American race, age category, prostate cancer Grade Group and the presence of cardiovascular disease were chosen a priori to serve as covariates in the analysis of our primary and secondary outcomes based on their known association with metastatic disease and ADT use.

### Statistical Analysis

The percentage of African American and non-African American men with distantly metastatic prostate cancer receiving ADT in combination with abiraterone was compared using the chi-squared test. Univariable logistic regression was used to determine the association between African American race and receipt of ADT in combination with abiraterone. We followed up with a multivariable model accounting for additional covariates (prostate cancer Grade Group, age category, and cardiovascular disease). Finally, the number of days from clinical stage change to distant metastatic disease to receipt of ADT with abiraterone was analyzed using negative binomial regression, first with just race, then with all covariates. All statistical analyses were performed in R Version 4.3.3 (R Foundation Vienna, Austria).

## Results

Within the PURC cohort, 201 of 1160 (17.3%) men with known race had documented distantly metastatic disease. A higher proportion of African American men had metastatic disease (57 of 257 [21%] vs. 144 of 903 [16%], *p* = 0.025) compared to non-African American men. Of those with metastatic disease, African American men were younger (27 of 57 [47%] vs. 40 of 144 [27.8%] age 69 or younger, *p* = 0.002) and had a higher proportion of cardiovascular disease (5 of 57 [8.8%] vs. 2 of 144 [1.4%], *p* = 0.021). Of the African American men with available health insurance payer data, a larger proportion of African American men had a private insurer (15 of 37 [39%] vs. 40 of 123 [32%]) or Medicaid (9 of 37 [24%] vs. 3 of 123 [2.4%]) as compared with non-African American men (*p* = 0.001). The median prostate specific antigen (PSA) at the time of metastatic disease diagnosis was 79 ng/ml in African American men compared to 28 ng/ml in non-African American men, though this difference was not statistically significant (*p* = 0.12). In addition, prostate cancer Grade Group, and the proportion of other co-morbid conditions were similar between the two groups (Table 1).

**Table 1:** Baseline characteristics of study cohort

	African American, *N* = 57	Non-African American, *N* = 144	*p*-value^1^
**Age range at diagnosis**, *n* (%)			0.005
59 and under	4 (7.0%)	4 (2.8%)	
60–69	23 (40%)	36 (25%)	
70–79	24 (42%)	52 (36%)	
80–89	5 (8.8%)	41 (28%)	
> 89	1 (1.8%)	11 (7.6%)	
**Ethnicity**, *n* (%)			0.2
Non-Hispanic	57 (100%)	133 (96%)	
Hispanic	0 (0%)	6 (4.3%)	
Unknown	0	5	
**BMI**, median (IQR)	27.3 (23.7–31.1)	27.4 (24.5–30.8)	0.7
Unknown	1	12	
**Primary insurance payer**, *n* (%)			0.001
Private	15 (39%)	40 (32%)	
Medicare	13 (34%)	71 (57%)	
Medicaid	9 (24%)	3 (2.4%)	
Self-pay	0 (0%)	2 (1.6%)	
VA	0 (0%)	2 (1.6%)	
Other	0 (0%)	5 (4.0%)	
Unknown	20	21	
**Highest education level**, *n* (%)			0.5
A high school diploma	5 (100%)	13 (62%)	
A college degree	0 (0%)	3 (14%)	
Some post-college or a graduate degree	0 (0%)	5 (24%)	
Unknown	52	123	
**Comorbidities**, *n* (%)			
History of myocardial infarction	4 (7.0%)	19 (13%)	0.2
History of chronic heart failure	2 (3.5%)	8 (5.6%)	0.7
History of peripheral vascular disease	0 (0%)	10 (6.9%)	0.065
History of cardiovascular disease	5 (8.8%)	2 (1.4%)	0.021
History of diabetes	4 (7.0%)	15 (10%)	0.5
History of diabetes with organ damage	3 (5.3%)	7 (4.9%)	> 0.9
**PSA at metastatic disease diagnosis**, median (IQR), ng/ml	79 (10–318)	28 (10–104)	0.12
Unknown	10	15	
**Grade group at diagnosis**, *n* (%)			0.6
Grade group 1–3	8 (14%)	15 (10.5%)	
Grade group 4–5	49 (86%)	128 (90%)	
Unknown	0	1	
**Year of clinical staging**, *n* (%)			0.10
2015	3 (5.3%)	4 (2.8%)	
2016	12 (21%)	15 (10%)	
2017	9 (16%)	17 (12%)	
2018	9 (16%)	15 (10%)	
2019	10 (18%)	33 (23%)	
2020	7 (12%)	19 (13%)	
2021	5 (8.8%)	35 (24%)	
2022	2 (3.5%)	6 (4.2%)	
**ADT usage**, *n* (%)	51 (89%)	135 (94%)	0.4
**LHRH type**, *n* (%)			0.4
Agonist	46 (81%)	126 (88%)	
Antagonist	5 (8.8%)	9 (6.3%)	
Other	6 (11%)	9 (6.3%)	
**ADT with abiraterone usage**	13 (23%)	43 (30%)	0.5

*ADT*, androgen deprivation therapy; *LHRH*, luteinizing hormone releasing hormone

^1^*p*–values were calculated using Fisher’s exact test for Count Data with simulated *p*-value (based on 2000 replicates); Fisher’s exact test; Wilcoxon rank sum test; Pearson’s chi-squared test

**Fig. 1:**
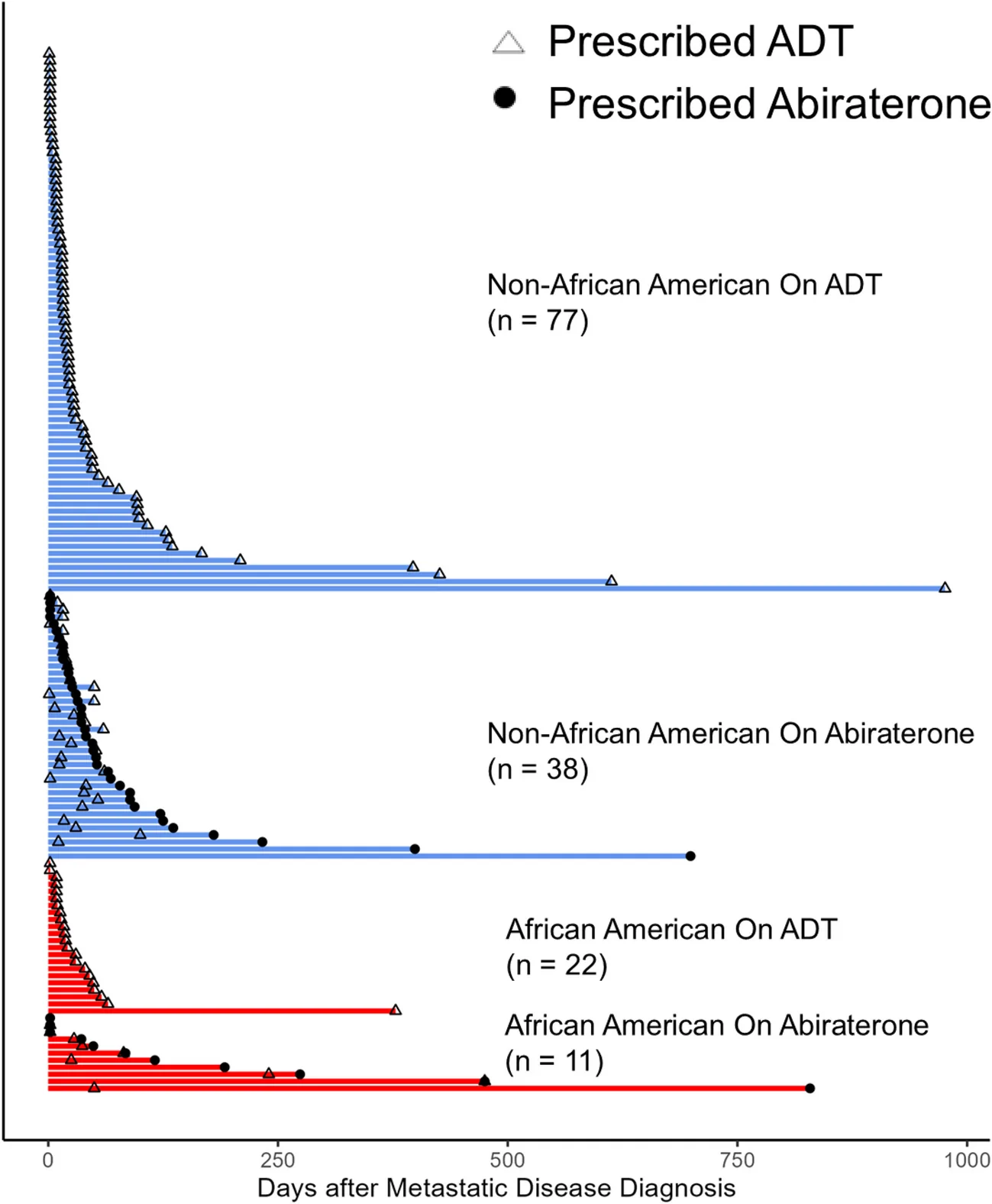
Time to initiation of abiraterone after diagnosis of metastatic disease cancer.^1^

Nearly all patients with distantly metastatic disease were prescribed ADT and the proportions of patients prescribed ADT were similar between African American and non-African American men (51 of 57 [89%] vs. 135 of 144 [94%], *p* = 0.46). Of these, 53 of 201 (26%) were prescribed ADT with abiraterone and the proportions were similar between African American and non-African American men (13 of 57 [23%] vs. 40 of 144 [28%], *p* = 0.5) (Table 1). In addition, there was no difference in the number of days between the diagnosis of metastatic disease and the initiation of ADT with or without abiraterone between African American and non-African American men (59.29 vs. 51.87 days,* p* = 0.61). However, the time between diagnosis of metastatic disease and initiation of abiraterone after beginning ADT was longer by 108 days for African American men compared to non-African American men (mean 187 vs. 79 days,* p* = 0.042) based on univariable negative binomial regression (Fig. 1). In addition, African American men with distantly metastatic disease experienced longer times between their initial prostate biopsy that diagnosed their cancer and initiation of ADT with abiraterone based on univariable negative binomial regression (predicted mean 226 vs. 91 days, *p* = 0.002).

On multivariable analysis, using race, age category, prostate cancer Grade Group, and cardiovascular disease, the difference in the predicted mean number of days between distantly metastatic disease diagnosis and ADT with abiraterone maintained the trend of African American men experiencing a longer time than non-African American men, but became non-significant (predicted mean 147 vs. 78 days, *p* = 0.17). However, age became a significant predictor with older patients (age > 89) initiating ADT with abiraterone sooner than the youngest patients (age ≤ 59) (mean 13.47 vs. 133.95 days, *p* = 0.036) after adjusting for race, prostate cancer Grade Group, and presence of cardiovascular disease (Tables 2 and 3)

**Table 2:** Predicted days between initiation of ADT with abiraterone from multivariable model based on covariates

	Predicted time (days, 95% confidence interval)	*p*-value^1^
**Race**		
Non-African American	77.53 (51.68–116.31)	Reference
African American	146.81 (65.75–327.82)	0.168
**Age category**		
≤ 59	133.95 (39.76–451.28)	Reference
60–69	78.12 (32.86–185.73)	0.474
70–79	118.32 (69.31–202.01)	0.851
80–89	68.01 (32.58–141.95)	0.350
> 89	13.47 (2.27–79.70)	0.036
**Grade group**		
Grade group 1–3	78.40 (30.91–198.83)	Reference
Grade group 4–5	91.81 (62.44–135.00)	0.757
**Cardiovascular disease**		
No	89.68 (63.16, 127.34)	Reference
Yes	79.95 (6.72, 950.85)	0.926

**Table 3:** Odds ratios from multivariable model for receipt of ADT with Abiraterone after metastatic disease diagnosis

	Odds ratio (95% confidence interval)	*p*-value
**Race**		
African American	Reference	Reference
Non-African American	1.46 (0.68–3.26)	0.344
**Age category**		
≤ 59	Reference	Reference
60–69	0.141 (0.023–0.696)	0.019
70–79	0.239 (0.041–1.13)	0.079
80–89	0.157 (0.025–0.800)	0.030
> 89	0.098 (0.009–0.757)	0.036
**Grade group**		
Grade group 2–3	Reference	Reference
Grade group 4–5	0.763 (0.293–2.15)	0.590
**Cardiovascular disease**		
No	Reference	Reference
Yes	0.406 (0.019–2.85)	0.440

## Discussion

In our study, we demonstrate in a large regional cohort that African American men with metastatic prostate cancer experience a delay in the initiation of ADT with abiraterone by approximately 3 months compared to their counterparts. African American men trended towards longer times between a diagnosis of distantly metastatic disease and abiraterone after controlling for prostate cancer grade group, age and history of cardiovascular disease though this was not statistically significant. Delays in time to ADT initiation alone among African American men with metastatic prostate cancer prior to the advent of ADT intensification have been reported previously in a population-based study by Keating et al. In their study, they showed that 12.7% of African American men in the Surveillance, Epidemiology, and End Results (SEER) database experienced delayed initiation of ADT alone by more than 4 months compared to 6.3% of White men (*p* < 0.001) [14]. In our study, the timing for the initiation of ADT alone among African American men was similar to non-African Americans; however, this timing diverged with the initiation of abiraterone. Thus, we highlight the potential lag time for dissemination of new agents among African American men. Prior reports have shown that novel agents in other cancer types such as systemic therapy for leukemia are often expanded to the African American population in a delayed fashion; however, this phenomenon is poorly studied in prostate cancer [15, 16]. A study by Ma et al. reported lower utilization of novel hormonal agents used in ADT intensification in the SEER database [16]. In that study, African American men with de novo metastatic prostate cancer were less likely to receive novel hormonal therapies like abiraterone in conjunction with ADT compared to White men. Moreover, this disparity persisted after adjusting for patient comorbidities, disease characteristics, and socioeconomic factors [16]. While the clinical benefit of ADT intensification on long-term survival in metastatic prostate cancer patients is well known, the long-term oncologic implications of delays in ADT intensification are less defined [9].

Thus, our study provides new information regarding ADT intensification administration by reporting disparities in the timing of its initiation. This level of granularity in describing gaps in metastatic prostate cancer care between African American and non-African American men receiving second-generation anti-androgens, to our knowledge, has not been documented previously. Potential mechanisms to explain the delay in abiraterone initiation that we have reported among African American men include distribution of insurance coverage. A prior study has shown that the use of patient assistance programs to help cover the out-of-pocket cost for abiraterone can be associated with a median delay of 30 days between the time drug prescription and receipt of the drug by the patient. In that study, the majority of patients requiring a patient assistance program to fill their abiraterone prescription had Medicare part D coverage [17]. In our study, we found that 35% of African American men with available insurance coverage data had Medicare for their health insurance; however, data regarding the use of financial assistance for abiraterone prescriptions are absent in the PURC database. As a result, it remains uncertain whether insurance payer contributes to the differential timing for the initiation of abiraterone in African American men in our cohort. Thus, further investigation is warranted in a larger cohort to clarify this.

The oncologic impact of delays in ADT intensification initiation in patients with metastatic prostate cancer are understudied with many reports evaluating the rate of use alone [16]. However, given that the time between metastatic disease diagnosis and ADT intensification in prior studies leading to its approval in clinical practice was 3 months or less than, it is conceivable that initiation beyond this time could be clinically relevant [9]. Thus, our study highlights an important gap in the granular detail surrounding the administration of second-generation anti-androgens. Potential future directions in this realm include investigating the timing of ADT intensification as an advanced prostate cancer care quality metric given its increased use in clinical practice and its absence as a key quality measure in prior studies [18].

### Limitations

While our study provides important data on a potential racial disparity in the timing of abiraterone initiation, it has some limitations. First, its retrospective design resulted in some amount of missing data which could lead to selection bias. Second, abiraterone was the only second-generation anti-androgen that was documented in the PURC database which limited our analysis of the use of other commonly used anti-androgens such as enzalutamide and apalutamide. Third, the PURC database did not collect information on testosterone level at the time that abiraterone was prescribed nor was information regarding whether abiraterone was prescribed for castrate sensitive or castrate-resistant metastatic disease provided which prevented evaluation of further granular data regarding administration of abiraterone. Fourth, data regarding the use of local therapy in the form of surgery or radiation prior to documented distantly metastatic disease was missing for the majority of patients which prevented us from identifying patients who developed metastatic disease after local treatment failure. Fifth, data regarding referral patterns after diagnosis of metastatic disease are absent from the PURC database, and potential transitions of care could contribute to differences in administration of abiraterone. Lastly, only a subset of patients in the PURC database developed metastatic disease within the follow-up period, leaving a small sample size for analysis. However, despite these limitations, strengths of our study and the PURC database include the regional and geographic diversity of the included patients as well as multi-institutional design which improves the generalizability of our findings.

## Conclusion

We demonstrate in a prospectively maintained multicenter regional cohort that while the use of abiraterone in combination with ADT in this cohort was similar between African American and non-African American men, the time between diagnosis of metastatic disease and abiraterone initiation was disproportionately delayed by 3 months in African American men. The clinical significance of this discrepancy warrants an in-depth investigation of system level barriers to timely initiation of abiraterone in African American men given the known benefits of abiraterone in combination with ADT. Future measures to mitigate the known disparities in metastatic prostate cancer care between racial groups will require in-depth evaluation of the granular differences in the administration of these life-prolonging agents.

## Data Availability

Data is unavailable due to closure of the PURC database on December 31, 2024.
